# Upregulated CD177 on neutrophils is implicated in sepsis pathogenesis and necroptosis-driven inflammation

**DOI:** 10.3389/fimmu.2026.1785356

**Published:** 2026-07-03

**Authors:** Haibo Liu, Xiangshu Cheng, Jun Li, Wei Wei, Yijia Yuan, Wenjuan Wang, Wei Wang, Fang Liu, Jin Zheng

**Affiliations:** 1 Department of Hematology, The First Affiliated Hospital of Xi’an Jiaotong University, Xi’an, Shaanxi, China; 2 School of Medicine, Northwest University, Xi’an, Shaanxi, China; 3 Department of Clinical Laboratory, The First Affiliated Hospital of Xi’an Jiaotong University, Xi’an, Shaanxi, China; 4 The Comprehensive Breast Care Center, The Second Affiliated Hospital of Xi’an Jiaotong University, Xi’an, Shaanxi, China; 5 The First Affiliated Hospital of Xi’an Jiaotong University, Xi’an, Shaanxi, China; 6 Department of Hematology, Ninth Hospital of Xi’an, Xi’an, Shaanxi, China; 7 Department of Renal Transplantation, Hospital of Nephrology, The First Affiliated Hospital of Xi’an Jiaotong University, Xi’an, Shaanxi, China

**Keywords:** CD177, necroptosis, neutrophil, nitric oxide, sepsis

## Abstract

**Introduction:**

Sepsis remains a leading cause of mortality in critical care, with dysregulated inflammatory responses driving disease progression. However, the role of necroptosis in sepsis pathogenesis remains incompletely understood.

**Methods:**

Here, through integration of multi-center cohort data (n = 1,265) and weighted gene co-expression network analysis (WGCNA), we constructed a six-gene necroptosis signature (CEBPD, CEBPB, MARCKS, SOCS3, PIM3, and JUNB) that correlated with sepsis severity and outcomes. Subsequently, we detected the expression of these genes in whole blood using qPCR. Furthermore, machine learning models incorporating this signature were evaluated across independent cohorts. Single-cell data analysis and flow cytometric analysis were further performed to characterize CD177^+^ neutrophils in sepsis.

**Results:**

All six Model-score genes were upregulated in sepsis patients, with four of them showing significant differences. Machine learning models incorporating this signature achieved robust diagnostic performance across independent cohorts. At the transcriptomic level, necroptosis activation showed a strong correlation with both the IL-6/STAT3 and TNF-α/NF-κB inflammatory pathways and distinct myeloid subsets. Single-cell data analysis further revealed that CD177^+^ neutrophils were significantly enriched in non-surviving sepsis patients and exhibited the highest necroptosis transcriptional score. Flow cytometric analysis revealed a significant increase in neutrophils, particularly the CD177^+^ subset, in the whole blood of septic patients. Furthermore, CD177^+^ neutrophils displayed blunted interferon responses alongside heightened production of inflammatory mediators, with nitric oxide (NO) potentially serving as an associated factor.

**Discussion:**

Collectively, our findings suggest that CD177+ neutrophils may be involved in necroptosis-related inflammation in sepsis and provide a clinically relevant gene signature for patient stratification, offering new perspectives for potential therapeutic exploration in sepsis management.

## 1 Introduction

Sepsis, a life-threatening condition characterized by organ dysfunction resulting from a dysregulated host response to infection (1), remains a major global healthcare challenge. Its most severe manifestation, septic shock, is marked by profound circulatory failure, cellular dysfunction, and metabolic derangements, leading to substantially increased mortality risk (2). The pathogenesis of sepsis involves complex molecular and cellular mechanisms, including dysregulated inflammatory responses, immune system perturbations, coagulation abnormalities, mitochondrial dysfunction, and altered autophagy pathways, collectively contributing to progressive organ failure (3). While recent advances in sepsis management have reduced mortality rates from 30-50% to 15-25% (4, 5), sepsis and septic shock remain leading causes of critical illness worldwide. The persistent high morbidity, coupled with the emergence of antimicrobial resistance, underscores the urgent need for innovative therapeutic approaches and novel drug development strategies to combat increasingly resistant pathogens.

Within the complex landscape of cell death mechanisms, necroptosis emerged as a distinct form of regulated inflammatory cell death, functioning as an alternative pathway when apoptosis is compromised (6). This programmed cell death process can be initiated through both extracellular death receptor signaling and intracellular pathogen detection pathways. Morphologically, necroptosis is characterized by distinctive features including organelle swelling, plasma membrane disruption, and cellular content release (7). The molecular machinery governing necroptosis centers on a well-defined signaling cascade involving TNF-α, Caspase-8, RIPK1, RIPK3, and MLKL (8). The execution of necroptosis leads to the release of cellular contents, serving as danger signals that provoke robust inflammatory responses and immune cell recruitment. Accumulating evidence has established necroptosis as a critical regulator in various pathological conditions, ranging from ischemic injury and neurodegeneration to cancer, viral infections, and autoimmune disorders (9). In infectious diseases, this cell death pathway interfaces with pathogen virulence mechanisms, as microorganisms deploy various factors to either trigger or evade host cell death responses.

In the context of sepsis, necroptosis has emerged as a crucial mediator of disease progression. RIPK3, a key molecular switch in the necroptotic pathway, has been validated as a significant prognostic indicator in clinical sepsis studies (10, 11). Further investigations have revealed complex dynamics of necroptotic signaling in sepsis, with elevated cellular expression of MLKL and RIPK3 observed in sepsis survivors, despite unchanged serum markers of necroptosis (12). Pharmacological intervention using Necrostatin-1 (Nec-1) to inhibit RIPK1 demonstrated therapeutic potential in experimental models, reducing pro-inflammatory cytokines (IL-6, IL-1β, IL-18) and chemokine expression while improving survival outcomes in neonatal sepsis (13). Intriguingly, necroptosis exhibits context-dependent effects in sepsis pathogenesis. While Nec-1-mediated necroptosis inhibition reduces RIPK1, RIPK3, and MLKL expression in the cecal ligation and puncture (CLP) model, it paradoxically exacerbates liver injury and decreases survival (14). Recent molecular insights have identified the miR-425-5p/RIPK1 regulatory axis as a potential protective mechanism against sepsis-induced hepatic damage (15), highlighting the complex role of necroptosis in organ dysfunction.

The advent of single-cell RNA sequencing (scRNA-seq) technology has revolutionized our understanding of cellular heterogeneity in sepsis. This powerful approach has enabled the identification of distinct transcriptional programs and intercellular communication networks in septic lung tissue, revealing potential therapeutic targets for sepsis-induced acute lung injury (16). Single-cell profiling has revolutionized our understanding of sepsis pathobiology by uncovering disease-specific cellular states and molecular signatures (17). Integration of scRNA-seq with multi-omics approaches has identified novel diagnostic markers, including MS1 signatures, while revealing the complex immune landscape in sepsis patients (18). However, the mechanistic understanding of these cellular phenotypes, particularly the role of MS1 cells and their regulatory networks in sepsis initiation, requires further investigation using experimental models.

Machine learning (ML) approaches have emerged as powerful tools for analyzing complex biological datasets. These computational methods excel at uncovering hidden patterns and relationships within large-scale data (19), as evidenced by their successful application in cancer research for diagnosis, prognosis, and treatment optimization (20). The integration of ML with biological data analysis offers unprecedented opportunities for understanding complex diseases like sepsis.

Here, we present an integrated analytical framework combining single-cell transcriptomics with sophisticated machine learning approaches to investigate necroptosis-associated gene networks in sepsis. By leveraging both single-cell and bulk RNA sequencing data, we identified differential gene expression patterns and co-expression networks linked to necroptotic pathways. Our comprehensive analytical pipeline incorporates multiple state-of-the-art ML algorithms, including K-nearest neighbor, support vector machines, and neural networks, to identify and prioritize potential risk genes in sepsis pathogenesis. These computational predictions were validated through qPCR analysis. This systematic integration of computational and experimental methods provides robust insights into sepsis mechanisms. This study establishes a novel framework for understanding necroptosis-mediated pathways in sepsis and identifies promising therapeutic targets, potentially paving the way for more effective treatment strategies in sepsis management.

## 2 Materials and methods

### 2.1 Data collection and processing

We obtained four publicly available datasets related to sepsis from the Gene Expression Omnibus (https://www.ncbi.nlm.nih.gov/): GSE13904 (normal=18, sepsis=158) (21), GSE54514 (normal=36, sepsis=127) (22), GSE65682 (normal=42, sepsis=760) (23), and GSE95233 (normal=22, sepsis=102) (24) are gene microarray datasets, while GSE167363 (normal=2, sepsis=10) (25) is a single-cell transcriptome dataset (**Supplementary Table 1**). The gene-gene interaction network for necrotic apoptosis was obtained from Zou et al. (26). For microarray data integration, we merged the GSE13904, GSE54514, GSE65682, and GSE95233 datasets and removed batch effects using ComBat algorithm. Subsequently, we extracted expression data for both normal control samples and sepsis samples from each dataset. A differential expression analysis was performed using the “limma” package to identify significantly altered genes between normal and sepsis samples. The expression differences of necroptosis-related genes were visualized using the “pheatmap” package. To better understand the biological roles of these genes in sepsis progression, we conducted enrichment analyses using the Kyoto Encyclopedia of Genes and Genomes (KEGG) and Gene Ontology (GO) databases through the Metascape online platform. The enrichment results were further visualized using Cytoscape to construct comprehensive pathway networks.

### 2.2 Single-cell RNA sequencing analysis

We analyzed the GSE167363 dataset containing single-cell transcriptome data from 2 normal individuals and 10 sepsis patients using the Seurat package (27). A stringent quality control pipeline was implemented: cells with mitochondrial gene content exceeding 10% were excluded, and only genes expressed in at least three cells with expression values ranging from 200 to 5000 were retained for downstream analysis. Batch effects between samples were eliminated using the Harmony package. Cell clustering was performed using Seurat’s FindClusters and FindNeighbors functions with default parameters, followed by dimensional reduction and visualization using Uniform Manifold Approximation and Projection (UMAP). Cell type annotation was conducted using SingleR (28) in conjunction with our curated marker gene set. For cell-composition comparisons, the proportion of each cell population was first calculated within each individual sample/donor. Group-level differences in cell-type proportions between normal and sepsis samples were then evaluated using sample-level proportions rather than treating individual cells as independent observations. Statistical comparisons were performed using the Wilcoxon rank-sum test with Bonferroni correction for multiple comparisons. Cell type proportions were visualized through bar graphs, and marker gene expression patterns across different cell populations were displayed using heatmaps.

### 2.3 Construction of necroptosis-related gene-set scores

To systematically evaluate necroptosis activity at the single-cell level, we developed a scoring system using single-sample Gene Set Enrichment Analysis (ssGSEA) (29). We first curated a comprehensive list of necroptosis-related genes from literature and databases, with detailed gene information provided in **Supplementary Table 2**. The expression levels of these genes were used to calculate a Necroptosis-related Gene-set Score for each cell. Based on the median Necroptosis-related Gene-set Score value, cells were stratified into High Necroptosis-related Gene-set Score and Low Necroptosis-related Gene-set Score groups to facilitate comparative analyses. Differential gene expression analysis between these groups was performed using the limma package, with a significance threshold of P<0.05. The resulting differentially expressed genes were carried forward for subsequent weighted gene co-expression network analysis.

### 2.4 Weighted gene co-expression network analysis

To identify gene modules associated with necroptosis in sepsis, we performed weighted gene co-expression network analysis (WGCNA) (30). First, we calculated Necroptosis-related Gene-set Score values in the merged microarray matrix using ssGSEA. The WGCNA analysis was conducted using the R package “WGCNA” with carefully optimized parameters: soft-thresholding power of 8 to ensure scale-free topology, maximum block size of 5000 genes to manage computational demands, minimum module size of 20 genes to focus on biologically meaningful modules, and module merging height threshold of 0.15 for appropriate granularity. We identified gene modules showing strong correlation with Necroptosis-related Gene-set Score and integrated these findings with differential expression analysis between normal and sepsis patients (threshold: P<0.05, |Logfc|>0.5) to identify key regulatory genes.

### 2.5 Construction of sepsis diagnostic model

Before classifier construction, candidate feature discovery was performed using the merged microarray datasets. Briefly, four microarray cohorts were integrated and batch-corrected using ComBat, followed by differential expression analysis and WGCNA. Genes located in the WGCNA salmon module, which was highly correlated with the necroptosis-related gene-set score, were intersected with sepsis-related differentially expressed genes to identify candidate features. The final six genes selected from this feature-discovery process were CEBPD, CEBPB, MARCKS, SOCS3, PIM3, and JUNB. These six candidate Model-score genes were then used as the fixed feature set for subsequent classifier construction and model comparison.

We developed a comprehensive machine-learning framework comprising 99 distinct models spanning multiple algorithmic approaches: logistic regression, linear and quadratic discriminant analysis, k-nearest neighbors (KNN), decision tree, random forest, XGBoost, ridge regression, lasso regression, elastic net regression, support vector machine (SVM), gradient boosting machine, stepwise logistic regression, and naive Bayes. For classifier construction, GSE95233 was used as the training cohort for model fitting and hyperparameter tuning, whereas GSE13904 and GSE54514 were used to evaluate model performance. During classifier training and validation, preprocessing was performed independently within each cohort without cross-dataset batch correction. Model optimization was performed using the caret package, which enabled systematic parameter tuning, customized parameter combinations, and cross-validation. The six Model-score genes and all optimized hyperparameters were locked before evaluation in the validation cohorts. Model performance was evaluated using both F-score and accuracy metrics. The final Model score was calculated using a weighted approach:


$$Model\;score=\;\sum_{i=1}^{n}\left(Importance_{i}*x_{i}\right)$$

where *Importance_i_* represents each gene’s model-determined importance and *x_i_* denotes its expression value.

### 2.6 Single-cell model-score and cell-composition analysis

To evaluate the distribution of the Model score across immune-cell populations, we calculated a single-cell Model-score module using AddModuleScore based on the six Model-score genes. The resulting scores were then compared across major immune-cell types and myeloid subclusters. To assess the distribution preference of each immune-cell subset across clinical groups, we calculated the ratio of observed to expected cell numbers (Ro/e). The observed value represented the actual number of cells from a given cell subset within each clinical group, whereas the expected value was calculated based on the overall frequency of that subset across all samples. Ro/e values greater than 1 indicated relative enrichment of a cell subset in a given group, whereas values less than 1 indicated relative depletion. For sample-level cell-composition comparisons, the proportion of each cell population was first calculated within each individual sample/donor, and group-level differences were then evaluated using these sample-level proportions rather than treating individual cells as independent observations.

### 2.7 Immune feature analysis

We performed immune cell deconvolution analysis using CIBERSORT (31) to estimate peripheral blood immune-cell composition. The input matrix was the normalized whole-blood gene-expression matrix generated after probe annotation, gene-symbol collapsing, and batch correction as described above. When multiple probes mapped to the same gene symbol, probe-level values were collapsed at the gene-symbol level before downstream analysis. CIBERSORT was run in relative mode using the LM22 leukocyte signature matrix to estimate the relative fractions of 22 immune-cell populations. Because all included transcriptomic datasets were generated from microarray platforms, quantile normalization for array data was enabled (arrays = TRUE). The analysis was performed with 100 permutations, and samples with CIBERSORT deconvolution P< 0.05 were retained for downstream immune-cell composition comparisons and correlation analyses. Differences in immune-cell fractions between high and low Model score groups were assessed using the Wilcoxon rank-sum test. Correlations between the six Model-score genes, the Model score, and immune-cell fractions were assessed using Spearman correlation analysis.

For immune-related pathway analysis, curated immune-related gene sets in GMT format were collected from published pathway resources and literature. Pathway activity scores were calculated for each sample using ssGSEA implemented in the GSVA package with Gaussian kernel estimation and absolute ranking. Differential pathway activity between high and low Model score groups was assessed using t-tests. The relationships between the six Model-score genes and immune-related pathway scores were further examined using Mantel’s test to evaluate gene-pathway associations.

### 2.8 Calculation of cytokine activity using CytoSig

Cytokine activity scores were computed using the Cytosig algorithm, which infers cytokine signaling activity based on the expression of downstream target genes (32). CytoSig analysis was performed on the Neu_CD177 cluster to compare cytokine signaling activity patterns among healthy controls, survived sepsis patients, and non-survived sepsis patients. The CytoSig framework provides Coef, StdErr, Zscore, and Pvalue as output metrics. In this study, the results were interpreted according to the corresponding P values, whereas z-scores were used to reflect the direction and relative magnitude of signaling activity rather than as a threshold for result selection.

### 2.9 Clinical sample collection and qPCR validation

We recruited 20 intensive care unit (ICU) patients for experimental validation, including 10 septic patients meeting Sepsis-3 criteria and 10 non-septic controls. Strict exclusion criteria were applied: age < 18 years, ICU stay < 24 hours, pregnancy or breastfeeding, malignancy, immunosuppressive therapy, and AIDS diagnosis. Whole blood samples were collected and processed within 2 hours using RNAiso Plus reagent (TaKaRa, Tokyo, Japan) following the manufacturer's protocol. cDNA synthesis was performed using the US Everbright reverse transcription kit (Suzhou, China). Quantitative PCR was conducted on an Applied Biosystems QuantStudio™ 3 system using Universal SYBR Green qPCR SuperMix. The amplification protocol consisted of initial denaturation (95°C, 10 minutes) followed by 40 cycles of denaturation (95°C, 15 seconds) and annealing/extension (60°C, 1 minute). Gene expression was analyzed using the 2-ΔΔCt method with GAPDH as the internal control. All primer sequences were validated for specificity and efficiency prior to use. The following primer sequences were used for qPCR validation: GAPDH forward: 5’- GTCAAGGCTGAGAACGGGAA-3’, reverse: 5’- AAATGAGCCCCAGCCTTCTC-3’; CEBPD forward: 5’-AGCAACGACCCATACCTCAGAC-3’, reverse: 5’-CTCGCAGTTTAGTGGTGGTAAGTC-3’; CEBPB forward: 5’- AGCCTCTCCACGTCCTCCTC-3’, reverse: 5’- GTGCTTGTCCACGGTCTTCTTG-3’; MARCKS forward: 5’- CCATTCTCTTGTCATTCAGGTCCAG-3’, reverse: 5’- GCCAAGCACTAAGCAATTACAATAGC-3’; SOCS3 forward: 5’-CATCTCTGTCGGAAGACCGTCA-3’, reverse: 5’-GCATCGTACTGGTCCAGGAACT-3’; PIM3 forward: 5’- CCGTCGACACTCTGTTTGGA-3’, reverse: 5’- CCCACCTGGTACGCCTTCTC-3’; JUNB forward: 5’- TCTACCACGACGACTCATACACAG-3’, reverse: 5’- GGCTCGGTTTCAGGAGTTTGTAG-3’; All primer sequences were obtained from the OriGene database (https://www.origene.com/), and the majority have been previously reported and validated in published studies.

### 2.10 Flow cytometric analysis of CD177^+^ neutrophils

Independent of the qPCR validation cohort described above, a separate cohort of 9 patients with sepsis and 9 healthy controls was recruited for flow cytometric analysis at a different time point. Therefore, the qPCR and flow cytometry datasets were interpreted as complementary rather than paired validation evidence. Patients with sepsis were recruited from the First Affiliated Hospital of Xi’an Jiaotong University, all of whom met the diagnostic criteria for sepsis. Sepsis was defined according to the most recent Sepsis-3criteria (33). Informed consent was obtained from all study participants

Peripheral blood samples were collected using EDTA anticoagulant tubes. For staining, 100 μL of fresh, gently mixed anti-coagulated whole blood was aliquoted into the bottom of a flow cytometry tube. Subsequently, 1 mL of 1× red blood cell lysis buffer (Proteintech, Cat. No: PF00014) was added to lyse red blood cells. The mixture was gently pipetted to resuspend the cells and incubated at 4°C for 5 minutes. Following lysis, the sample was centrifuged after adding a threefold volume of PBS. The supernatant was discarded.

The cell pellet was then stained for 30 minutes at 4°C in the dark with the following fluorescently conjugated anti-human antibodies: APC/Fire™750-conjugated anti-CD45 (BioLegend, Cat: 304062), APC-conjugated anti-CD15 (BioLegend, Cat: 301907), PE-conjugated anti-CD16 (BioLegend, Cat: 302007), and FITC-conjugated anti-CD177 (BioLegend, Cat: 315803). After staining, cells were washed and resuspended for analysis.

All samples were analyzed using a CytoFlex flow cytometer (Beckman Coulter, USA). Data acquisition was performed with CytExpert software (Beckman Coulter), followed by analysis using FlowJo software (version 10.9). The gating strategy included sequential gates for stringent quality control: initial debris exclusion using an FSC-A vs. SSC-A gate, followed by SSC-H vs. SSC-A gating to eliminate dead cells and doublets. Single-cell purity was further ensured using an FSC-H vs. FSC-A gate to exclude remaining doublets and cellular aggregates. Within the live singlet population, CD45^+^ leukocytes were identified and gated for subsequent analysis. Fluorescence-minus-one (FMO) controls were established for all key markers to define accurate gating boundaries, and full compensation matrices were applied during data acquisition to correct for spectral overlap. The levels of CD15^+^CD16^+^ neutrophils and CD177^+^ neutrophils were identified and quantified according to the gating strategy established for CD45^+^ leukocytes.

### 2.11 Western blotting analysis

For Western blot analysis, cells were lysed in NP-40 lysis buffer (150 mM NaCl, 1.0% NP-40, and 50 mM Tris-HCl, pH 8.0) supplemented with protease and phosphatase inhibitor cocktails. The lysates were sonicated and centrifuged at 12,000 × g for 10 min to remove insoluble debris, and the supernatants were collected. Protein concentrations were determined using a BCA Protein Assay Kit (Beyotime, cat. no. P0012) according to the manufacturer’s instructions. Briefly, 20 μL of each protein sample was mixed with 200 μL of BCA working reagent, and protein concentrations were calculated based on a standard curve. Protein samples were then mixed with loading buffer and denatured at 95 °C for 5 min. Equal amounts of protein were separated by SDS-PAGE and transferred onto PVDF membranes. The membranes were blocked with 5% skim milk for 1 h at room temperature and incubated overnight at 4°C with primary antibodies against anti-RIPK3 (phospho-S227) (Cohesion Biosciences, cat. no. CPA6622), recombinant anti-MLKL (phospho-S345) (Cohesion Biosciences, cat. no. CMA9335), and GAPDH (Immunoway, cat. no. YM3029). After primary antibody incubation, the membranes were washed three times with TBST for 10 min each and then incubated with HRP-conjugated goat anti-rabbit IgG (Boster, cat. no. BA1054) or HRP-conjugated goat anti-mouse IgG (Boster, cat. no. BA1050) for 1 h at room temperature. The membranes were subsequently washed three additional times with TBST for 10 min each. Protein bands were visualized using an enhanced chemiluminescence detection system (Baygene Biotech, China), and images were captured using a gel imaging system with appropriate exposure times to avoid signal saturation. The relative abundance of each target protein was quantified as the ratio of its band intensity to that of the corresponding GAPDH.

### 2.12 Data visualization and reproducibility

We generated comprehensive visualizations using the ggplot2 package in R. Heatmaps were created using the pheatmap package with row-wise standardization and hierarchical clustering where appropriate. For network visualization, we employed Cytoscape to display gene-gene interactions and pathway relationships. Single-cell data visualizations were generated through Seurat's built-in functions, with custom color schemes applied to enhance clarity. All statistical plots indicate significance levels using standardized notation (* p < 0.05, ** p < 0.01, *** p < 0.001).

### 2.13 Statistical analysis

All statistical analyses were conducted using R version 4.1.0. For comparing continuous variables between groups, we employed either Student's t-test or Mann-Whitney U test depending on data distribution characteristics as determined by the Shapiro-Wilk normality test. When analyzing categorical variables, we utilized Chi-square test or Fisher's exact test based on expected cell frequencies. Multiple testing correction was performed using the Benjamini-Hochberg method to control false discovery rate, with statistical significance consistently defined as p < 0.05 unless otherwise specified.

For differential expression analysis in both bulk and single-cell data, we implemented empirical Bayes moderation through the limma package to improve the reliability of variance estimates. Correlation analyses between gene expression, immune cell populations, and pathway activities were conducted using Spearman’s rank correlation coefficient, with significance assessed after multiple testing correction. The evaluation of cell type distribution differences between normal and sepsis samples employed the Wilcoxon rank-sum test with Bonferroni correction.

### 2.14 Statistical analysis

All statistical analyses were conducted using R version 4.1.0. For comparing continuous variables between groups, we employed either Student’s t-test or Mann-Whitney U test depending on data distribution characteristics as determined by the Shapiro-Wilk normality test. When analyzing categorical variables, we utilized Chi-square test or Fisher’s exact test based on expected cell frequencies. Multiple testing correction was performed using the Benjamini-Hochberg method to control false discovery rate, with statistical significance consistently defined as p< 0.05 unless otherwise specified.

For differential expression analysis in both bulk and single-cell data, we implemented empirical Bayes moderation through the limma package to improve the reliability of variance estimates. Correlation analyses between gene expression, immune cell populations, and pathway activities were conducted using Spearman’s rank correlation coefficient, with significance assessed after multiple testing correction. The evaluation of cell type distribution differences between normal and sepsis samples employed the Wilcoxon rank-sum test with Bonferroni correction.

## 3 Results

### 3.1 Integrated analysis of multiple sepsis microarray datasets reveals significant alterations in necroptosis pathways

**Figure 1:**
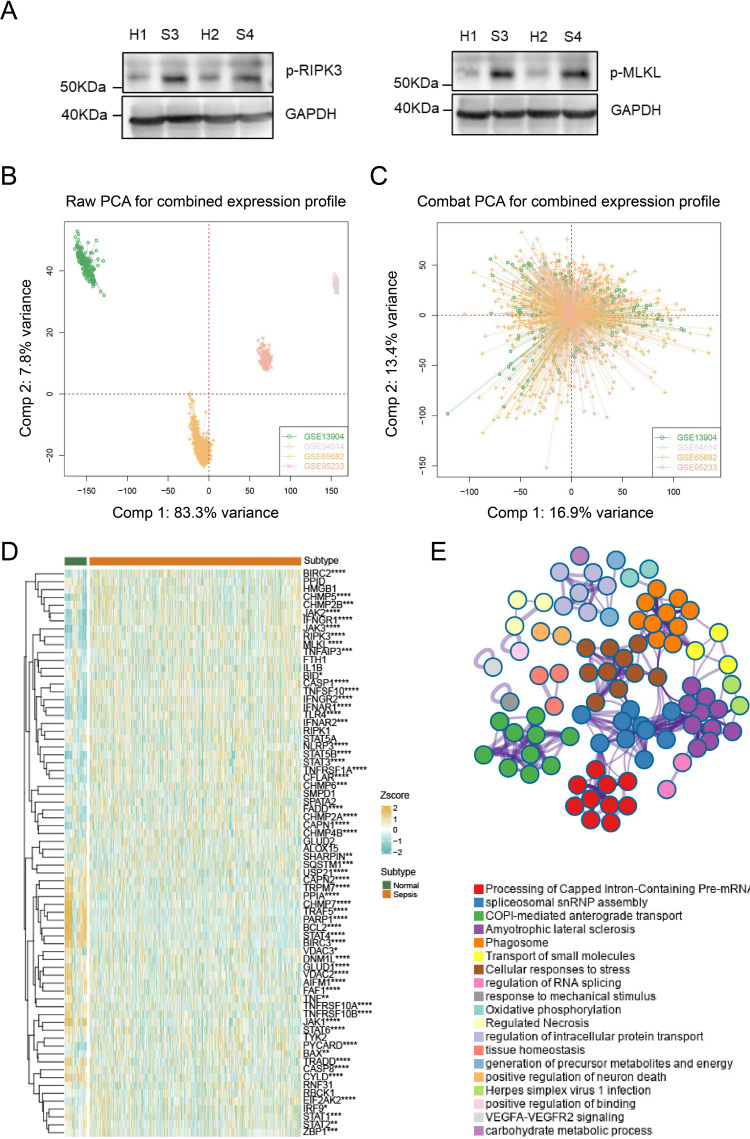
Evaluation of necroptosis-related transcriptional features and protein markers, and integrated analysis of multiple sepsis microarray datasets. **(A)** Western blot analysis of necroptosis-related protein markers, p-RIPK3 and p-MLKL, in peripheral blood samples from sepsis patients and healthy controls. **(B)** Principal component analysis (PCA) plot of four independent sepsis microarray datasets (GSE13904, GSE54514, GSE65682, and GSE95233) before batch-effect correction. **(C)** PCA plot of the same samples after batch-effect correction using the ComBat algorithm. **(D)** Heatmap showing the expression patterns of necroptosis-related genes in sepsis patients and healthy controls. **(E)** Metascape pathway enrichment analysis of differentially expressed necroptosis-related genes.

Since systemic hyperinflammation in sepsis can trigger necroptosis, we evaluated its status in patient whole blood cells. Western blotting demonstrated significantly higher levels of p-RIPK3 and p-MLKL in sepsis patients than in healthy controls (**Figure 1A**), confirming the activation of the necroptotic pathway during sepsis. To systematically investigate molecular alterations in sepsis, we first conducted an integrated analysis of four publicly available sepsis transcriptome datasets. These datasets, comprising GSE13904, GSE54514, GSE65682, and GSE95233, collectively encompassed 1,147 sepsis patients and 118 healthy controls. Principal component analysis initially revealed distinct batch effects among these datasets (**Figure 1B**). To ensure analytical reliability, we successfully eliminated these batch effects using the Combat algorithm, generating a high-quality harmonized dataset suitable for comprehensive analysis (**Figure 1C**).

Using this integrated dataset, we performed differential expression analysis between sepsis patients and healthy controls. Through systematic pathway enrichment analysis, we discovered that necroptosis-related pathways emerged as one of the most significantly altered biological processes. This finding led us to thoroughly examine the expression patterns of genes involved in necroptosis regulation. Hierarchical clustering analysis clearly demonstrated distinct expression signatures of necroptosis-related genes between sepsis patients and healthy controls (**Figure 1D**).

Further pathway analysis using Metascape revealed that these differentially expressed genes were significantly enriched in several critical biological processes, including the processing of capped intron-containing pre-mRNA, phagosome formation, and regulated necrosis (**Figure 1E**). These results suggest that necroptosis-related transcriptional alterations may be associated with sepsis and motivated subsequent analyses of necroptosis-related transcriptional features in this disease context.

### 3.2 Co-expression network analysis reveals key gene modules associated with necroptosis and inflammation in sepsis

**Figure 2:**
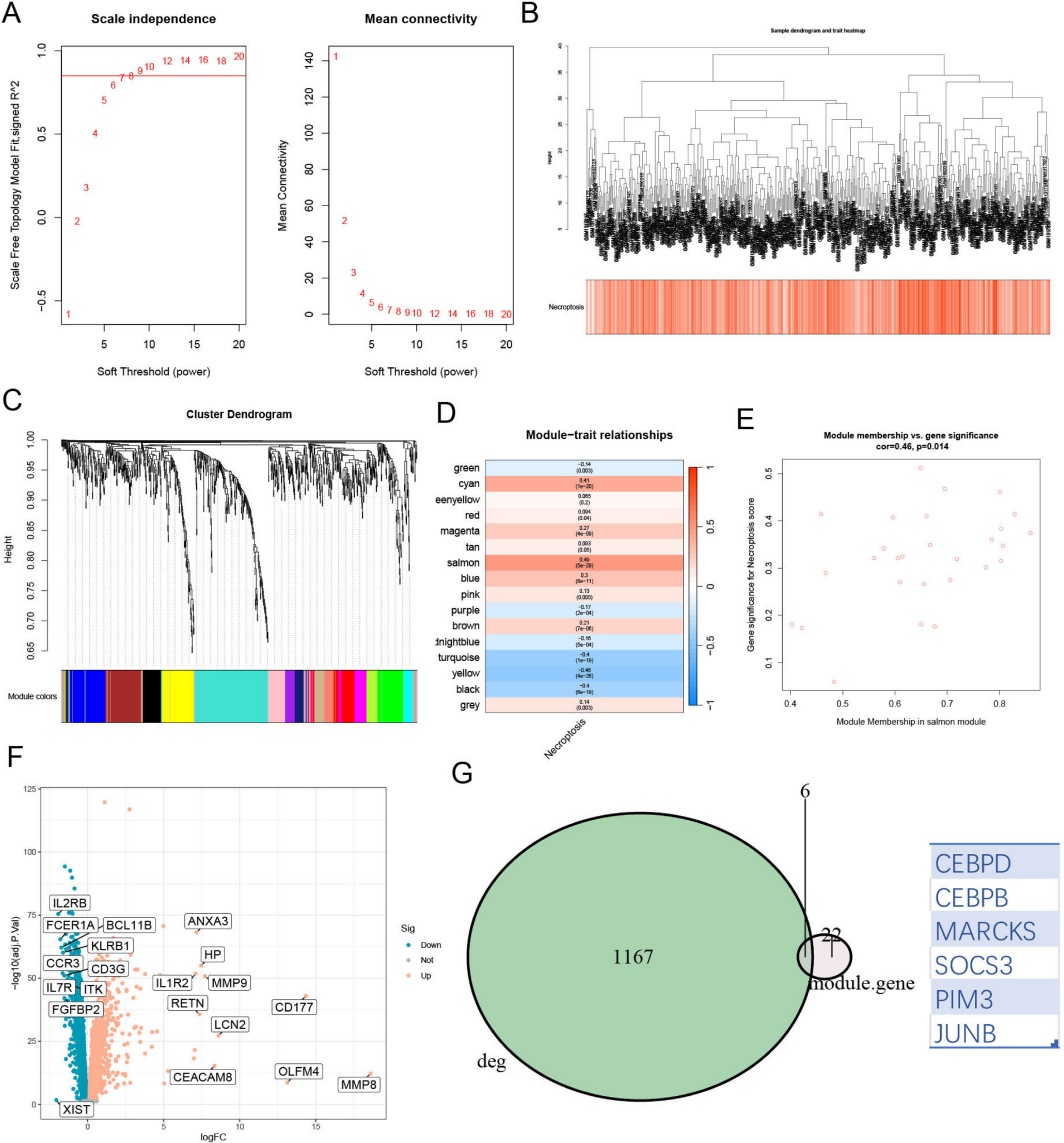
Integrated co-expression network and differential expression analyses identify candidate Model-score genes associated with necroptosis-related transcriptional features in sepsis. **(A)** Scale-free topology fit index and mean connectivity across soft-thresholding powers; power = 8 was selected for network construction. **(B)** Hierarchical clustering dendrogram of genes after quality control, with the necroptosis-related gene-set score shown below. **(C)** Gene modules identified using dynamic tree cutting, yielding 16 co-expression modules. **(D)** Module-trait correlation analysis between module eigengenes and the necroptosis-related gene-set score; the salmon module showed the strongest positive correlation. **(E)** Correlation between module membership and gene significance within the salmon module. **(F)** Volcano plot showing differentially expressed genes between sepsis patients and healthy controls, with selected inflammatory genes labeled. **(G)** Intersection of differentially expressed genes and salmon module genes identified six candidate Model-score genes: CEBPD, CEBPB, MARCKS, SOCS3, PIM3, and JUNB.

Given the complex interplay between necroptosis and inflammation in sepsis, we hypothesized that identifying co-expressed gene networks could identify gene modules associated with disease-related transcriptional features. To systematically map these gene-gene relationships, we employed weighted gene co-expression network analysis (WGCNA). First, we determined the optimal network parameters by analyzing soft threshold powers, which revealed that a power value of 8 achieved the best approximation of scale-free topology while maintaining high mean connectivity (**Figure 2A**). Based on these optimized parameters, we constructed a hierarchical clustering tree with distinct gene modules indicated by color coding (**Figure 2B**).

Using dynamic tree cutting methods, we clustered genes into 16 distinct co-expression modules, each marked with a unique color for identification (**Figure 2C**). Further investigation of module-trait relationships revealed that the salmon module exhibited the strongest positive correlation with necroptosis-related gene-set score (**Figure 2D**). Analysis of module membership within this module demonstrated significant positive correlation (correlation coefficient = 0.46, p = 0.014), suggesting that genes within this module may be associated with necroptosis-related transcriptional features (**Figure 2E**).

Differential expression analysis identified 1,167 differentially expressed genes, including several inflammatory mediators (**Figure 2F**). Comparison of these differential genes with those in the salmon module revealed six shared candidate Model-score genes: CEBPD, CEBPB, MARCKS, SOCS3, PIM3, and JUNB (**Figure 2G**). Notably, among the differentially expressed genes, we observed significant upregulation of CD177, a neutrophil-associated marker, along with inflammatory mediators MMP8 and MMP9, clearly visible in the volcano plot (**Figure 2F**). This finding suggests a potentially transcriptional association between neutrophil-mediated inflammatory responses and necroptotic pathways.

Together, the WGCNA and differential expression analyses identified a salmon co-expression module associated with the necroptosis-related gene-set score and yielded six candidate Model-score genes: CEBPD, CEBPB, MARCKS, SOCS3, PIM3, and JUNB. The upregulation of CD177, MMP8, and MMP9 further suggested that neutrophil-related inflammatory signals may be linked to these necroptosis-related transcriptional features, supporting subsequent analyses focused on neutrophil subsets.

### 3.3 Machine learning model development and experimental validation of necroptosis-associated diagnostic markers

**Figure 3:**
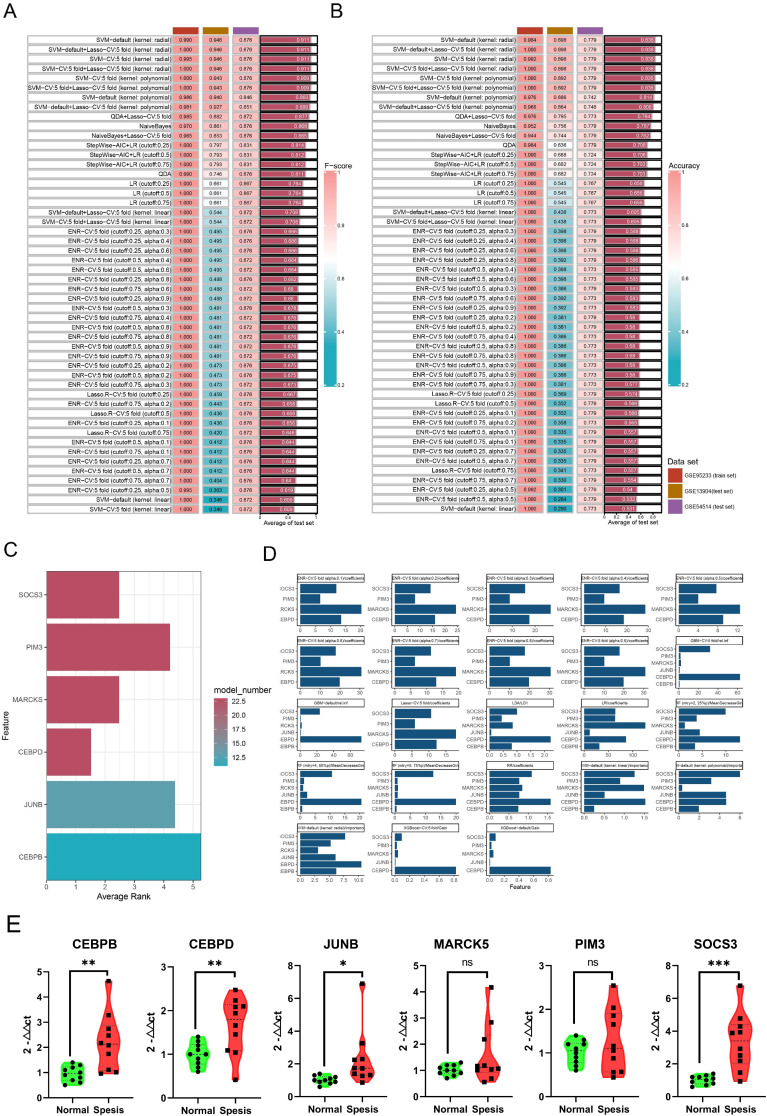
Machine-learning model development and experimental assessment of the six Model-score genes in sepsis. **(A)** Comparison of machine-learning model performance based on F-scores. The left panel shows model ranking by F-score, and the right panel shows average performance across datasets. Colors indicate different algorithm categories. **(B)** Comparison of model performance based on accuracy. The heatmap shows accuracy values across different models and datasets, with red indicating higher accuracy. **(C)** Average importance ranking of the six Model-score genes in the selected model. The bar plot shows the relative contribution of each gene to model classification performance. **(D)** Feature-importance analysis of the six Model-score genes across different machine-learning models. **(E)** qPCR assessment of the six Model-score genes in healthy controls (green, n = 10) and sepsis patients (red, n = 10). CEBPB, CEBPD, JUNB, and SOCS3 showed higher expression in the sepsis group, whereas MARCKS and PIM3 did not reach statistical significance (*p < 0.05, **p < 0.01, ***p < 0.001; ns, not significant).

Having identified six candidate genes (CEBPD, CEBPB, MARCKS, SOCS3, PIM3, and JUNB) through network analysis, we next sought to evaluate their clinical utility as diagnostic markers. Using these genes as features, we systematically evaluated multiple machine learning approaches using a comprehensive performance assessment framework. Through rigorous comparison of model performance metrics, we identified SVM with radial kernel (SVM-default) as the optimal algorithm, achieving the highest F-score, accuracy and Recall-score across different validation datasets (**Figures 3A, B**; **Supplementary Figure 1**).

Feature importance analysis revealed differential contributions of the six Model-score genes to model performance. SOCS3, PIM3, and MARCKS demonstrated particularly strong predictive power, while JUNB and CEBPB showed moderate but consistent contributions across multiple model implementations (**Figure 3C**). This pattern was further validated through independent evaluations across different modeling frameworks, confirming the robust predictive value of these markers (**Figure 3D**).

To validate the biological relevance of these computational findings, we performed qPCR analysis on blood samples from sepsis patients (n=10) and healthy controls (n=10). This experimental validation revealed significant upregulation of CEBPB (p< 0.01), CEBPD (p< 0.01), JUNB (p< 0.05), and SOCS3 (p< 0.001) in sepsis patients compared to controls. Notably, while MARCKS and PIM3 showed trends toward increased expression in sepsis samples, these changes did not reach statistical significance (**Figure 3E**). These results provided experimental support for altered expression of several Model-score genes in sepsis.

Together, the machine-learning analysis and qPCR validation suggest that the six Model-score genes may help distinguish sepsis patients from healthy controls at the transcriptomic level. Further validation in larger prospective cohorts is needed to determine their diagnostic utility and clinical applicability.

### 3.4 Pathway analysis reveals distinct biological programs associated with necroptosis status

**Figure 4:**
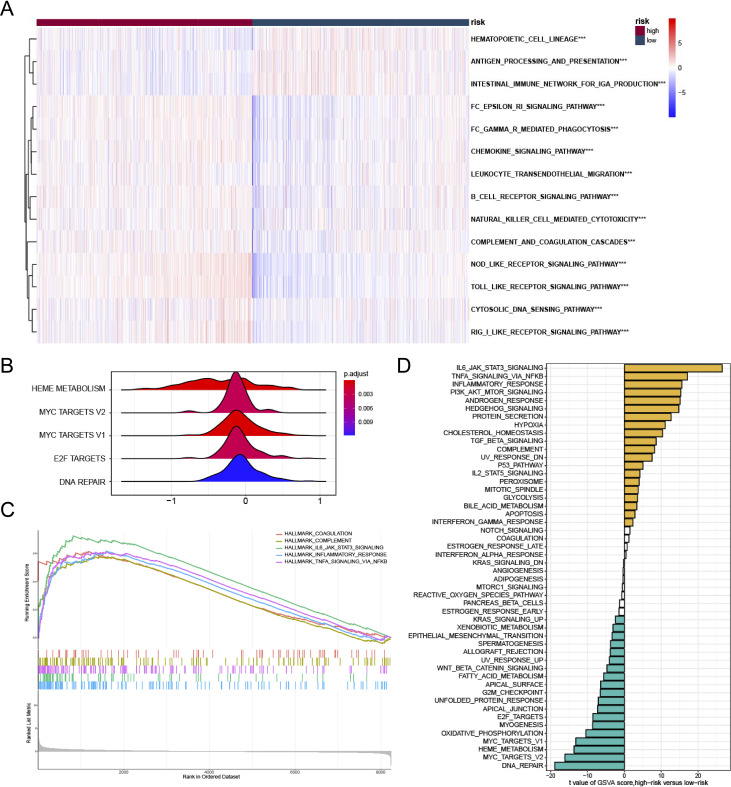
Integrated pathway analysis of biological programs associated with the Model score in sepsis. **(A)** Heatmap showing immune-related pathway scores between low- and high-Model-score groups. Low-Model-score samples showed higher scores for hematopoietic cell lineage and antigen processing pathways, whereas high-Model-score samples showed higher scores for pattern recognition receptor pathways, including NOD-like, Toll-like, and RIG-I-like receptor signaling. **(B)** Ridge plot showing enrichment of DNA repair and cell-cycle-related pathways, including MYC and E2F targets, in the low-Model-score group (adjusted p < 0.003). **(C)** Running enrichment score plots showing enrichment of inflammation-related pathways, including coagulation, complement, IL6-JAK-STAT3 signaling, and TNFα signaling, in high-Model-score samples. **(D)** GSVA analysis comparing pathway scores between low- and high-Model-score groups. Bar length indicates t-value magnitude; positive values indicate higher scores in high-Model-score samples, whereas negative values indicate higher scores in low-Model-score samples.

After evaluating the performance of the six Model-score genes and assessing their expression patterns, we next sought to understand the broader biological context associated with necroptosis-related transcriptional features in sepsis. Using multiple complementary analytical approaches, we first analyzed immune-related pathways curated from the KEGG database. Our heatmap analysis revealed distinct pathway activation patterns between patient groups. In the low-Model-score group, we observed significant enrichment of pathways related to hematopoietic cell lineage, antigen processing and presentation, and intestinal immune network for IgA production. In contrast, the high-Model-score showed enrichment of pattern recognition receptor pathways, including NOD-like receptor, Toll-like receptor, and RIG-I-like receptor signaling pathways, as well as cytosolic DNA sensing pathway (**Figure 4A**). This distribution pattern suggests differences in immune-related pathway scores between the groups.

To gain a more comprehensive view of biological processes, we performed Gene Set Enrichment Analysis (GSEA) using the 50 Hallmark gene sets. This analysis revealed a striking dichotomy in pathway activation. Ridge plot analysis clearly demonstrated that cell cycle and proliferation-related pathways, including DNA repair, E2F targets, and MYC targets, were significantly enriched in the low-Model-score group (**Figure 4B**). Conversely, the running enrichment score plots showed sustained enrichment of inflammation-related pathways in the high-Model-score group, including coagulation, complement activation, IL6-JAK-STAT3 signaling, inflammatory response, and TNFα signaling via NFκB (**Figure 4C**).

We further validated these findings using Gene Set Variation Analysis (GSVA). The results confirmed that inflammatory pathways (IL6-JAK-STAT3, inflammatory response, and TNFα signaling) consistently showed higher activation scores in the high-Model-score group, while cell cycle-related pathways (DNA repair and E2F targets) demonstrated stronger activity in the low-Model-score group (**Figure 4D**).

These results suggest an immune-related transcriptional pattern associated with the Model score: the low-Model-score group exhibited higher scores for adaptive immune-related pathways, while the high-Model-score group showed higher scores for innate immune recognition and inflammatory pathways. This systematic pathway analysis provides a transcriptomic basis for further exploring immune-related differences associated with the Model score in sepsis.

Notably, correlation analysis revealed that the six Model-score genes showed particularly strong associations with NOD-like receptor and Toll-like receptor signaling pathways. These pathways, in turn, demonstrated strong positive correlations with multiple other immune pathways, suggesting their potential involvement in coordinating the immune response in sepsis (**Supplementary Figure 2**). To validate the robustness of our findings, we examined the expression patterns of the six Model-score genes across all four independent datasets, confirming their consistent association with sepsis through odds ratio analysis and demonstrating their predictive accuracy using ROC curves (**Supplementary Figure 3**).

### 3.5 Immune cell composition analysis reveals myeloid cell enrichment in sepsis

Given the enrichment of innate immune recognition pathways in high-risk sepsis patients, we next investigated which immune cell populations might be driving these molecular signatures. Using CIBERSORT for computational deconvolution analysis of whole-blood transcriptomic data, we uncovered significant alterations in immune cell composition between sepsis patients and healthy controls. Most notably, we observed a marked increase in myeloid cell populations in sepsis patients, with neutrophils showing the most pronounced elevation, followed by significant increases in macrophage subsets and mast cells.

**Figure 5:**
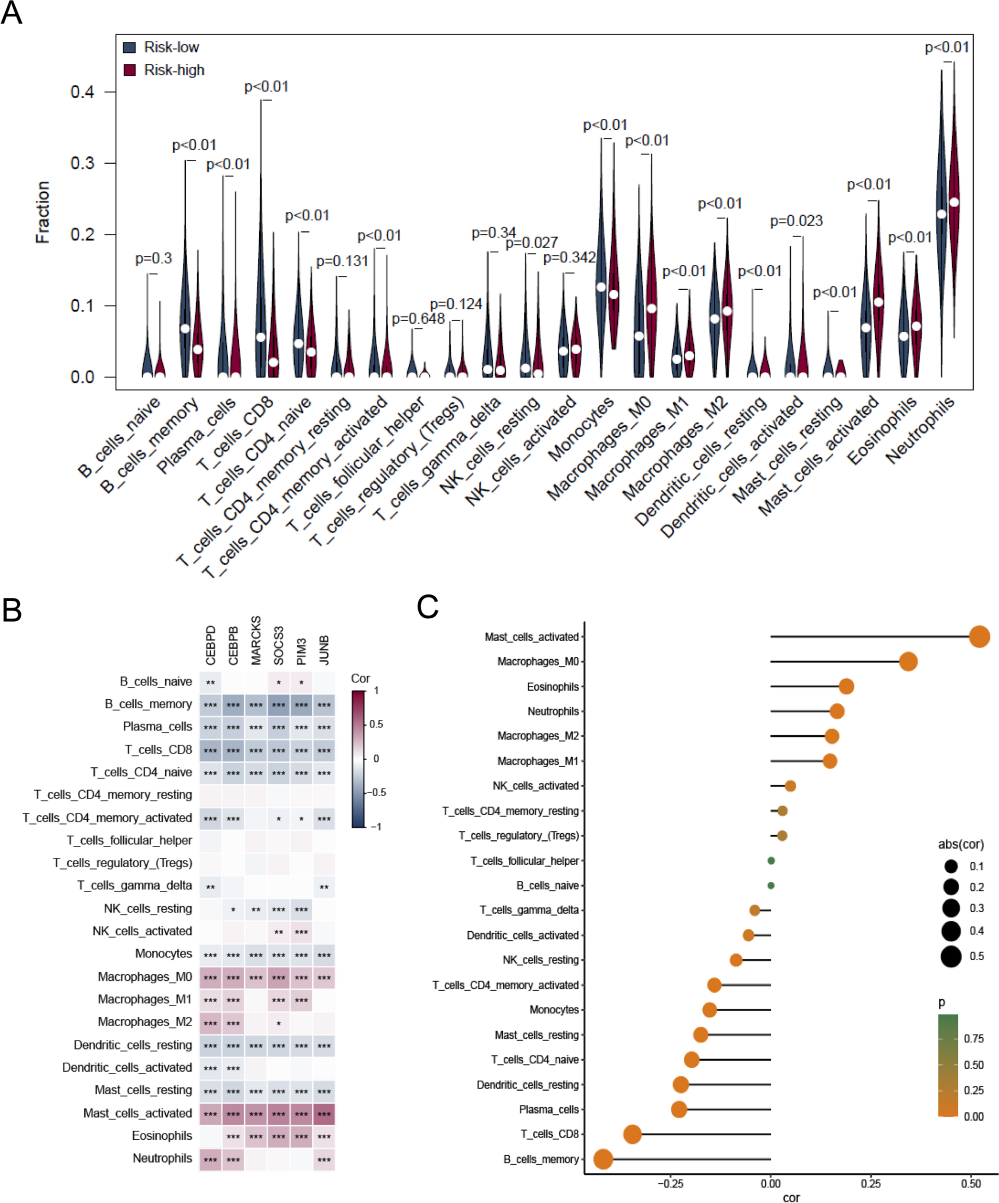
Estimated immune-cell composition associated with the Model score in sepsis whole-blood transcriptomic analysis. **(A)** Distribution of estimated immune-cell fractions between low- and high-Model-score groups. Violin plots with embedded box plots show differences in estimated myeloid cell fractions, including neutrophils, macrophage subsets, and mast cells, between groups. **(B)** Correlation analysis between the six Model-score genes and estimated immune-cell fractions. The heatmap shows Spearman correlation coefficients between gene expression levels and immune-cell fractions; red indicates positive correlations and blue indicates negative correlations. Statistical significance: *p < 0.05, **p < 0.01, ***p < 0.001. **(C)** Correlation analysis between the Model score and estimated immune-cell fractions. The lollipop plot shows correlation coefficients and statistical significance for each immune-cell type.

When examining the relationship between immune cell composition and the Model score, we found that samples with high Model scores showed higher estimated proportions of myeloid cells. Specifically, neutrophils demonstrated the strongest correlation with the Model score, while activated mast cells and different macrophage populations (M0, M1, and M2) also showed significant positive associations (**Figures 5A, C**).

Further analysis of the six Model-score genes revealed strong correlations with these expanded myeloid populations. The expression levels of the six Model-score genes showed robust positive associations particularly with neutrophils, macrophages, and activated mast cells (**Figure 5B**). These findings suggest that the expansion of myeloid cell populations, especially neutrophils, may be associated with sepsis-related transcriptional changes and higher Model scores.

### 3.6 Single-cell transcriptomics reveals distinct immune cell diversity patterns associated with sepsis outcomes

While our bulk RNA sequencing analysis revealed important shifts in immune cell populations, this approach cannot fully capture the heterogeneity of immune responses at the cellular level, nor distinguish between different activation states within the same cell type. To overcome these limitations and gain higher resolution insights into immune cell dynamics during sepsis progression, we performed single-cell RNA sequencing analysis. We first conducted rigorous quality control of our single-cell sequencing data. Through analysis of RNA feature counts, mitochondrial gene expression ratios, and hemoglobin gene expression levels, we ensured that data quality met analytical standards (**Supplementary Figure 4A**). UMAP visualization demonstrated good mixing of cells from different patient samples, indicating successful batch effect removal (**Supplementary Figure 4B**). Using cell type-specific marker gene expression profiles (**Supplementary Figure 4C**), we accurately identified and classified various immune cell subsets, confirming cell type distribution characteristics through quantitative statistics (**Supplementary Figure 4D**). Independent analysis of each patient showed consistent immune cell distribution patterns with no abnormal individual variations (**Supplementary Figure 4E**).

**Figure 6:**
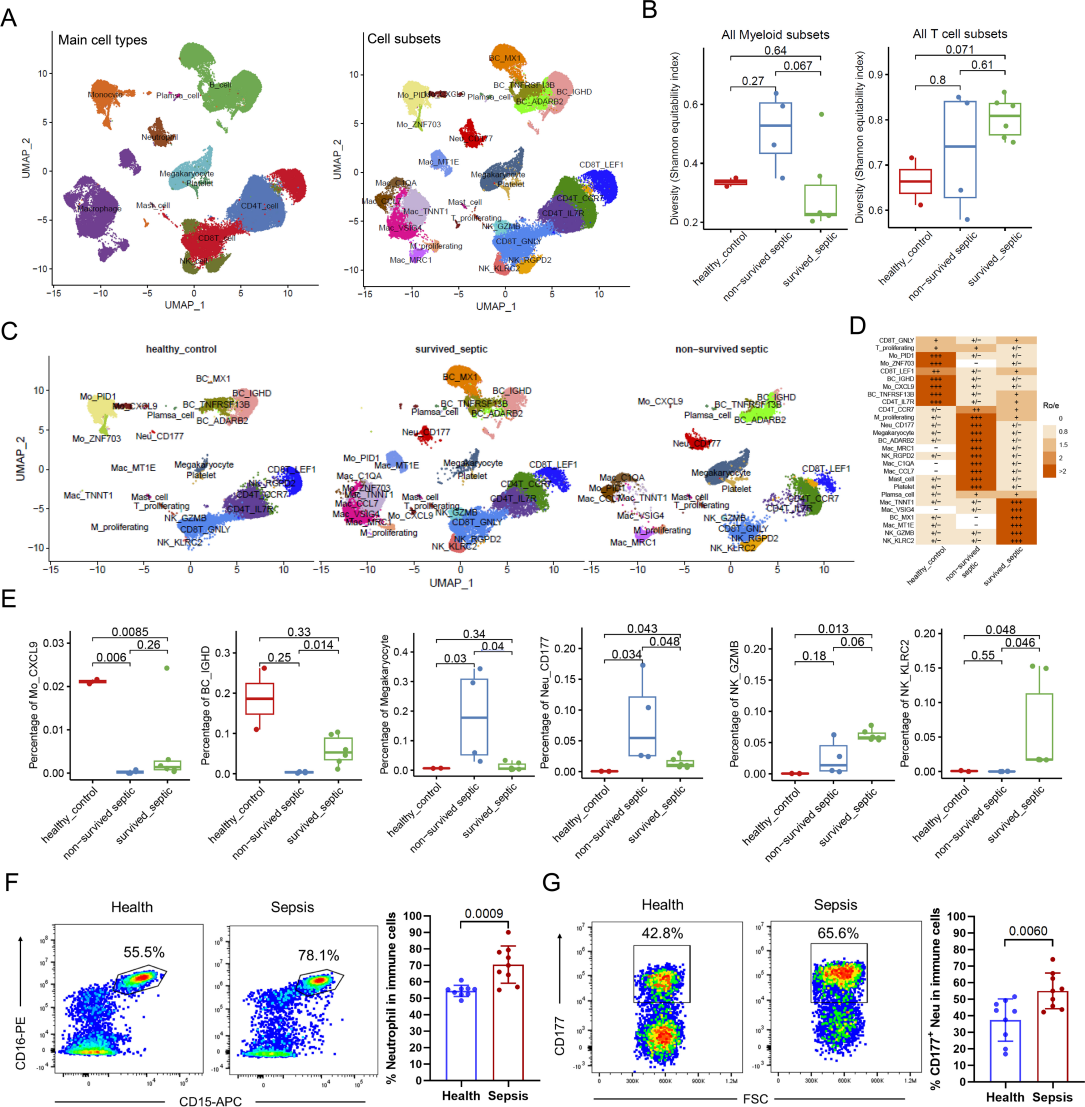
Single-cell transcriptomic analysis of immune-cell diversity and sample-level cell-population differences associated with sepsis outcomes. **(A)** UMAP visualization of immune-cell populations. The left panel shows major immune-cell types, and the right panel shows detailed immune-cell subsets. Each dot represents a single cell and is colored by cell-type annotation. **(B)** Quantification of immune-cell diversity across healthy controls, non-surviving septic patients, and surviving septic patients. The left panel shows myeloid-cell diversity, and the right panel shows T-cell diversity across groups. **(C)** UMAP plots showing immune-cell subset distributions across healthy controls, surviving septic patients, and non-surviving septic patients. **(D)** Ro/e-based analysis of cell-type distributions across groups. The heatmap shows relative enrichment or depletion of specific cell populations across clinical groups, with asterisks indicating statistical significance (*p < 0.05, **p < 0.01, ***p < 0.001). **(E)** Sample-level comparison of representative immune-cell subsets across groups. Box plots show the proportion of each cell subset within individual samples, including Mo_CXCL9, NK_KLRC2, and Neu_CD177. **(F)** Flow cytometric analysis of CD15^+^CD16^+^ neutrophil frequency in sepsis patients and healthy controls (p = 0.0009). **(G)** Flow cytometric analysis of CD177^+^ neutrophil frequency in sepsis patients and healthy controls (p = 0.0060).

Building on this robust analytical foundation, we performed in-depth comparisons across samples from healthy controls, surviving septic patients, and non-surviving septic patients. UMAP visualization clearly demonstrated the distribution of major immune cell types (**Figure 6A**, left panel) and their detailed subsets (**Figure 6A**, right panel). When examining immune cell diversity, we discovered a striking pattern (**Figure 6B**): severe sepsis patients exhibited higher myeloid cell diversity, potentially associated with uncontrolled inflammatory responses, while mild sepsis patients showed enhanced T cell diversity, a characteristic that may be associated with more effective pathogen clearance.

To gain more granular understanding, we compared immune cell subset distribution patterns across the three groups (**Figure 6C**) and quantitatively analyzed these distributions using the Ro/e algorithm (**Figure 6D**). Further analysis revealed several cell subsets showing group-specific enrichment (**Figure 6E**): Mo_CXCL9 was significantly elevated in healthy controls (p = 0.006), NK_KLRC2 was enriched in surviving septic patients (p = 0.046), and notably, Neu_CD177 showed a higher sample-level proportion in non-surviving septic patients (p = 0.034), suggesting this cell subset might be associated with poor disease outcomes. Peripheral blood samples from 9 sepsis patients and 9 healthy controls were collected and analyzed using flow cytometry (**Supplementary Figure 4F**). The analysis revealed a significant increase in the frequency of CD15^+^CD16^+^ neutrophils in sepsis patients compared to healthy controls (p = 0.0009) (**Figure 6F**). Additionally, a significant increase in the frequency of CD177^+^ neutrophils was observed in the sepsis group (p = 0.006) (**Figure 6G**).

These findings characterize changes in immune cell composition during sepsis progression and suggest distinct patterns of cell subsets associated with disease outcomes: increased myeloid cell diversity may be linked to dysregulated inflammation, while maintained T cell diversity may be associated with favorable disease outcomes. Through this analysis, we provide transcriptomic evidence of immune cell heterogeneity in sepsis, which may help prioritize future studies of immune-cell subsets in disease progression.

### 3.7 Cellular and molecular analysis reveals distinct inflammatory features of Neu_CD177 in sepsis

To characterize inflammatory transcriptional features in sepsis, we first conducted a systematic analysis of immune cell activation patterns. AddModuleScore analysis of 50 Hallmark pathways revealed higher inflammatory pathway scores in myeloid cells compared to other immune populations (**Supplementary Figure 5A**). This finding prompted us to examine the distribution of the Model score across major immune cell types, which notably showed the highest scores in neutrophils (**Supplementary Figure 5B**).

**Figure 7:**
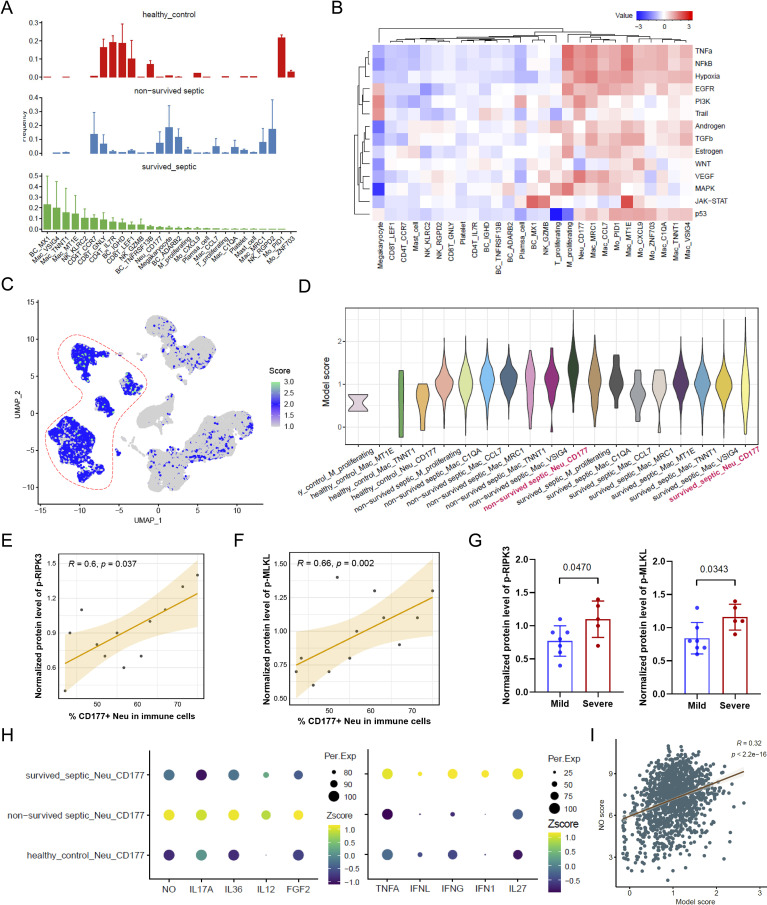
Neu_CD177⁺ neutrophils are associated with inflammatory features and necroptosis-related markers in sepsis. **(A)** Relative immune-cell proportions across healthy controls, non-surviving septic patients, and surviving septic patients. Bar plots show the distribution of immune-cell populations across patient groups. **(B)** Pathway activity analysis across immune-cell populations using PROGENy. The heatmap shows inferred pathway activity scores for 14 signaling pathways across annotated immune-cell subsets. **(C)** UMAP visualization of the single-cell Model score across immune-cell populations. Blue dots indicate cells with higher Model scores, and gray dots indicate other immune-cell populations. **(D)** Distribution of the single-cell Model score across annotated immune-cell subsets. Violin plots show Model-score distributions, with Neu_CD177 highlighted in red. **(E)** Correlation between the proportion of CD177^+^ neutrophils among immune cells and the protein level of p-RIPK3. **(F)** Correlation between the proportion of CD177⁺ neutrophils among immune cells and the protein level of p-MLKL. **(G)** Comparison of p-RIPK3 and p-MLKL protein levels between mild and severe sepsis patients. **(H)** CytoSig-based cytokine signaling analysis of Neu_CD177 cells across healthy controls, surviving septic patients, and non-surviving septic patients. Dot size indicates the percentage of cells expressing the indicated cytokine-response signature, and color indicates the scaled expression/activity score. **(I)** Correlation between NO-related signaling score and the single-cell Model score in neutrophils from septic patients.

To understand the functional implications of these findings, we first analyzed the cellular contributions to inflammation in sepsis. Quantitative comparison revealed significant differences in immune cell proportions, with myeloid subsets, showing the pronounced variation between groups (**Figure 7A**). Pathway analysis using Progeny further demonstrated heightened activation of inflammatory pathways, including TNF-α, NFκB, and TGF-β signaling, predominantly in myeloid subsets (**Figure 7B**).

Focusing on myeloid subsets, we further discovered that Neu_CD177 cells exhibited the highest Model score among the analyzed myeloid subsets (**Figures 7C, D**). To ensure the specificity of this finding and exclude potential interference from generic inflammatory signals, we performed additional validation analyses using independent, well-established gene sets (**Supplementary Table 2**). Using an established necroptosis gene set from public databases, we observed that necroptosis-related gene-set scores were also highest in Neu_CD177 cells (**Supplementary Figure 6**), consistent with our original findings. In contrast, analysis using the MSigDB HALLMARK_INFLAMMATORY_RESPONSE gene set showed no specific enrichment in Neu_CD177 cells (**Supplementary Figure 6**), suggesting that the elevated necroptosis-related score in Neu_CD177 cells was not solely attributable to generic myeloid inflammation. These validation analyses support the relative specificity of the necroptosis-related transcriptional signature. GSEA analysis revealed significantly impaired interferon responses (both IFN-γ and IFN-α) in neutrophils from non-surviving septic patients (**Supplementary Figure 5C**; NES = -1.87 and -1.86, p< 0.01), suggesting a potential association with reduced pathogen-clearance-related transcriptional programs. Furthermore, examination of the six Model-score genes showed higher expression in neutrophils from severe sepsis patients (**Supplementary Figure 5D**), particularly evident in non-surviving cases.

Subsequent analysis revealed a significant positive correlation between CD177+ neutrophil abundance and the expression of p-RIPK3 (**Figure 7E**) and p-MLKL (**Figure 7F**) in the peripheral blood of sepsis patients. Moreover, the expression of these necroptotic markers was significantly elevated in severe sepsis patients relative to those with mild disease (**Figure 7G**). Collectively, these results indicate that extensive necroptosis is associated with sepsis severity and may hinder disease control.

The inflammatory characteristics of Neu_CD177 were further explored by cytokine signaling analysis, which suggested increased inferred cytokine-response activity in non-surviving septic patients (**Figure 7H**). Notably, we observed a significant positive correlation between NO signaling score and the Model score in neutrophils (R = 0.32, p = 2.2e-16), suggesting a potential association between NO-related signaling and necroptosis-related transcriptional features in these cells (**Figure 7I**).

These integrated analyses suggest that Neu_CD177 is associated with dysregulated inflammatory transcriptional features in severe sepsis, characterized by higher necroptosis-related transcriptional scores, increased inferred inflammatory mediator activity, and reduced interferon-response signatures. This comprehensive analysis identifies a cell subset associated with sepsis severity and outcome-related transcriptional changes and provides candidate directions for future functional and translational studies.

## 4 Discussion

This study focuses on necroptosis related transcriptional features in sepsis and their associated molecular regulatory networks. By integrating bulk transcriptome data and single-cell sequencing data analysis, we systematically characterized the association of Neu_CD177 with necroptosis-related transcriptional features in sepsis progression. The main findings include: (1) multi-center cohort integration revealing increased expression of necroptosis-related genes in sepsis patients and the identification of candidate Model-score genes (CEBPD, CEBPB, MARCKS, SOCS3, PIM3, and JUNB) associated with necroptosis-related gene-set scores through WGCNA; (2) machine learning models supporting the potential diagnostic relevance of these six Model-score genes in sepsis, supported by qPCR experiments demonstrating significant differential expression of multiple genes; and (3) single-cell transcriptomic profiling showing a higher sample-level proportion of Neu_CD177 in patients with severe sepsis, associated with higher Model scores and suggesting a potential link between these cells and persistent inflammatory transcriptional features.

Recent advances in understanding necroptosis have revolutionized our perspective on programmed cell death in sepsis pathogenesis. Unlike apoptosis, which is generally immunologically silent, necroptosis triggers robust inflammatory responses through the release of damage-associated molecular patterns (DAMPs) (34). The necroptotic pathway, primarily regulated by RIPK1, RIPK3, and MLKL, has emerged as a critical determinant of sepsis outcomes (35). Previous studies have demonstrated that elevated RIPK3 levels correlate with increased mortality in septic patients (36), while MLKL activation drives tissue damage in experimental sepsis models (37). Our findings extend these observations by identifying Neu_CD177 as a neutrophil subset associated with necroptosis-related transcriptional features, suggesting a potential mechanism by which these cells may be linked to inflammatory responses through death receptor and pattern recognition receptor signaling pathways. This aligns with recent evidence showing that neutrophil necroptosis contributes to vascular dysfunction and organ failure in severe sepsis (38).

The six Model-score genes identified in our study represent candidate Model-score genes associated with sepsis-related transcriptional alterations. CEBPD and CEBPB, members of the CCAAT/enhancer-binding protein family, have been previously implicated in myeloid cell differentiation and inflammatory responses (39). Recent studies have shown that CEBPB regulates neutrophil function during acute inflammation (40), while CEBPD modulates innate immune responses in sepsis-induced acute lung injury (41). MARCKS, a protein kinase C substrate, plays essential roles in neutrophil migration and degranulation (42), while SOCS3 acts as a critical negative regulator of cytokine signaling (43). PIM3, a serine/threonine kinase, has been linked to cell survival and inflammatory responses (44), and JUNB regulates myeloid cell differentiation and inflammatory gene expression (45). Our identification of the coordinated expression of these genes in association with Neu_CD177 provides new insights into how these genes may be linked to necroptosis-related transcriptional features and inflammation in sepsis.

At the transcriptomic level, neutrophils serve as critical first responders in innate immunity, with their classical functions including phagocytosis and pathogen killing thoroughly documented (44, 45). Recent studies have demonstrated that dysregulated neutrophil activation in sepsis can trigger excessive release of neutrophil extracellular traps (NETs) and inflammatory mediators (35). Our findings extend this understanding by showing that higher necroptosis-related transcriptional scores in neutrophils, particularly in the Neu_CD177 subset, may represent a transcriptional feature associated with inflammatory amplification. This aligns with recent work showing that neutrophil necroptosis contributes significantly to organ dysfunction in severe inflammatory conditions (37, 38). The observed accumulation of Neu_CD177 in our study parallels findings from other severe inflammatory conditions where similar neutrophil subsets are associated with disease pathology (35, 46). Our discovery of strong correlations between necroptosis and classical inflammatory pathways (IL-6/STAT3 and TNF-α/NF-κB) builds upon previous observations in other disease models (46, 47), suggesting a potential association with inflammatory amplification.

From a clinical translational perspective, the six-gene Model score proposed here and its link to Neu_CD177 enrichment may have potential diagnostic and prognostic relevance. On one hand, combining this multigene Model score with machine learning algorithms may help identify patients likely to progress to severe disease, although further prospective validation is needed before clinical application. On the other hand, inhibiting key necroptotic mediators (e.g., RIPK1/RIPK3) or modulating Neu_CD177 function may represent candidate directions for future investigation, but these strategies require direct functional validation. Previous studies have shown partial success of certain small-molecule inhibitors (e.g., Necrostatin-1) or microRNA-based interventions (e.g., the miR-425-5p/RIPK1 axis) in animal models, though further large-scale clinical trials are needed before these strategies can be fully translated into clinical practice.

Nonetheless, several limitations of the present study should be acknowledged. First, although multi-cohort integration and single-cell sequencing provided a relatively comprehensive transcriptomic foundation, heterogeneity in patient populations, sample processing, and platform characteristics across different centers cannot be entirely excluded. Larger prospective cohorts are therefore needed for further independent validation. Second, our conclusions are primarily based on bioinformatic analyses and associative evidence, such as module scoring, pathway enrichment, and correlation analyses. Although we supplemented the study with Western blot detection of p-RIPK3 and p-MLKL, as well as correlation analyses linking these necroptosis-related markers with CD177+ neutrophils, the causal relationship between CD177+ neutrophils and necroptosis-related inflammation remains to be established. Further functional experiments, such as necroptosis inhibitor treatment, CD177 blockade, and assays using sorted CD177+ neutrophils, are still needed to directly validate the mechanistic involvement of CD177+ neutrophils in necroptosis-related pathways. Third, the role of NO-related signaling was inferred from correlation analyses, and direct experimental evidence, such as nitrite/nitrate measurement, NOS2 protein expression, or cellular NO assays, is needed to determine whether NO signaling functionally contributes to necroptosis-related inflammation. Fourth, although the classifier was trained in GSE95233 and evaluated in GSE13904 and GSE54514, future studies with larger sample sizes and additional independent cohorts are still needed to further validate the robustness and generalizability of the Model score based on the six Model-score genes, particularly at the feature-discovery stage. Ideally, such studies should use a fully independent discovery-training-validation framework, in which feature discovery, model training, and external validation are performed in non-overlapping cohorts. Finally, necroptosis may intersect with other forms of regulated cell death, such as pyroptosis and ferroptosis. As this study focused primarily on necroptosis-related transcriptional features, future research should incorporate multiple modes of programmed cell death into an integrated framework to better understand their collective association with multi-organ dysfunction in sepsis.

In summary, through an integrative approach combining molecular, cellular, bioinformatic, and machine learning methodologies, this study characterizes necroptosis-related transcriptional features in sepsis and identifies Neu_CD177 as a cell subset associated with pathological inflammation. The six-gene Model score developed herein may provide exploratory insight for early diagnosis and future translational studies in sepsis, laying a foundation for subsequent large-scale clinical and functional validations. Ultimately, more precise therapeutic strategies targeting necroptosis-related hyperinflammation will require further mechanistic and clinical validation before their potential impact on sepsis outcomes can be determined.

## Data Availability

The raw data supporting the conclusions of this article will be made available by the authors upon reasonable request, without undue reservation.
