# Supplementary material for: Upregulated CD177 on neutrophils is implicated in sepsis pathogenesis and necroptosis-driven inflammation
Source: Front Immunol. 2026 Jul 3;17:1785356. doi: 10.3389/fimmu.2026.1785356 (PMC13375507; doi:10.3389/fimmu.2026.1785356)
Supplement: Supplementary file 5 [file Supplementaryfile2.pdf]

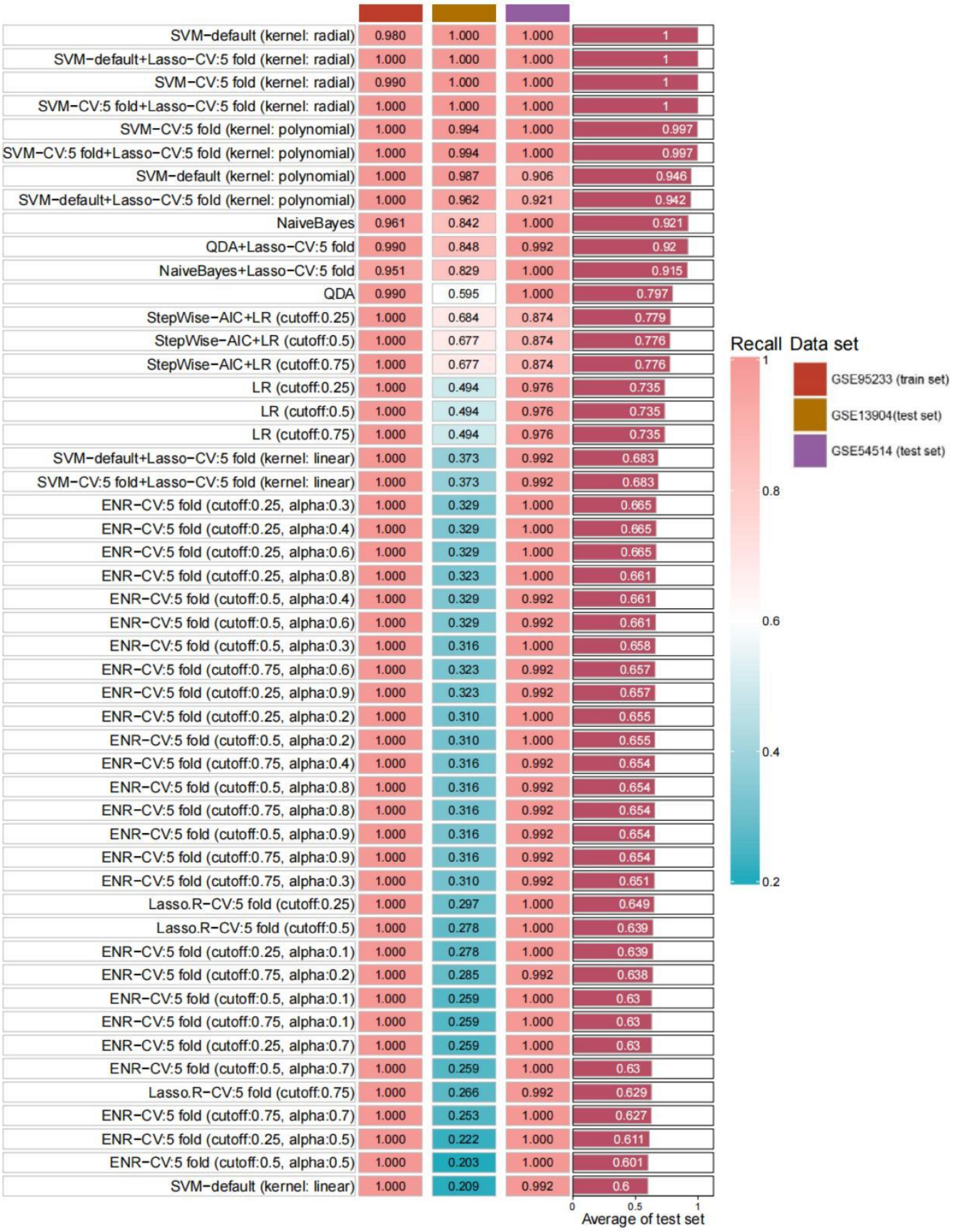

Supplementary Figure 1. Recall-based evaluation of machine-learning models.

Heatmap showing recall values for different machine-learning models across GSE95233 (training set), GSE13904 (test set), and GSE54514 (test set). The rightmost column shows the average recall across the two test sets. Color intensity indicates recall values, with red representing higher recall and blue representing lower recall..

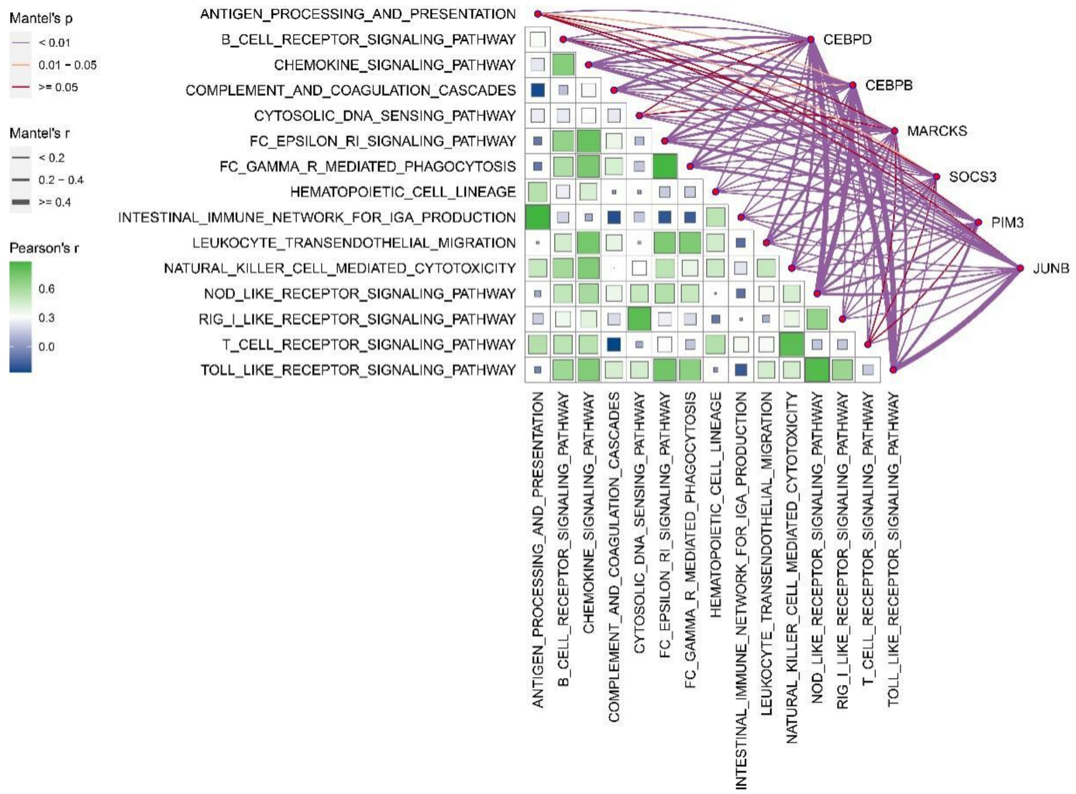

Supplementary Figure 2. Association analysis between the six Model-score genes and immune-related pathway scores.

Correlation and Mantel test analyses showing the relationships between the six Model-score genes and immune-related pathway scores derived from KEGG gene sets. The heatmap displays Spearman correlation coefficients among immune-related pathways, with green indicating positive correlations and blue indicating negative correlations. Curved lines indicate Mantel test associations between the six Model-score genes and immune-related pathways, with line color representing statistical significance and line width representing Mantel's r value. Statistical significance levels are indicated as *p < 0.05, **p < 0.01, and ***p < 0.001.

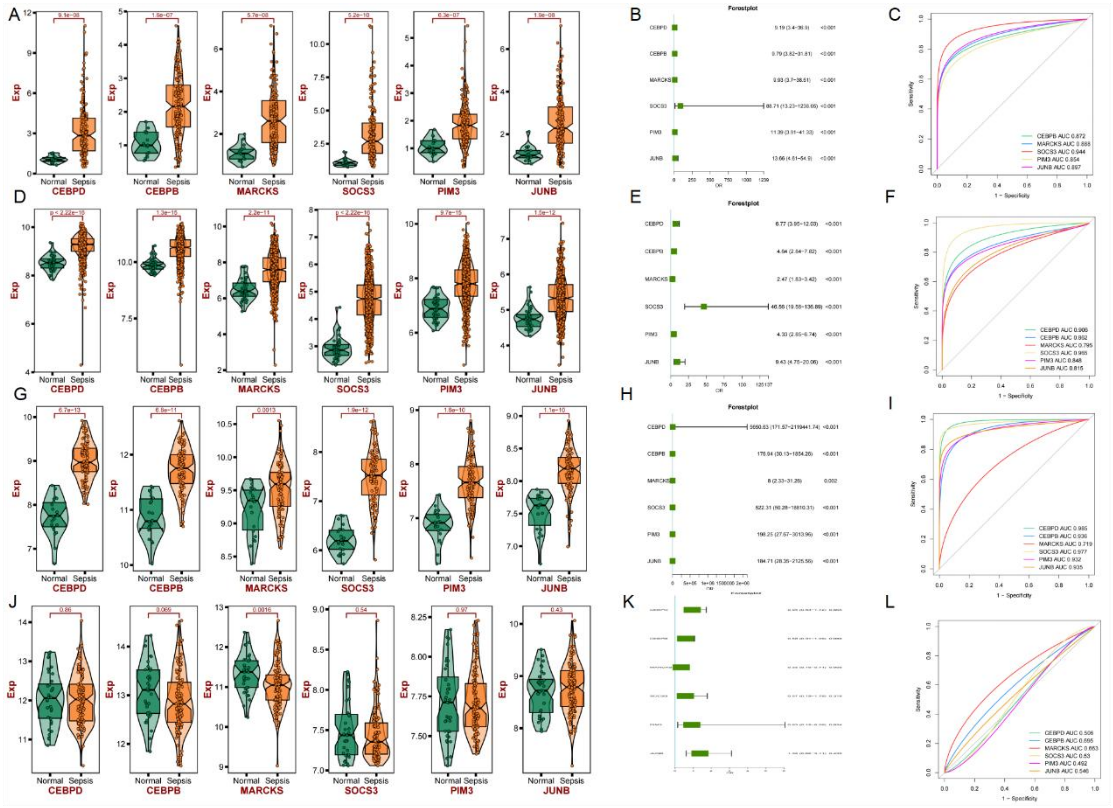

Supplementary Figure 3. Evaluation of the six Model-score genes across sepsis transcriptomic cohorts.

Expression patterns, odds ratio analyses, and ROC curves were used to evaluate the association between the six Model-score genes and sepsis status across four publicly available datasets (GSE13904, GSE54514, GSE65682, and GSE167363).

(A-C) Expression patterns, odds ratios, and ROC analysis in GSE13904.

(D-F) Expression patterns, odds ratios, and ROC analysis in GSE54514.

(G-I) Expression patterns, odds ratios, and ROC analysis in GSE65682.

(J-L) Expression patterns, odds ratios, and ROC analysis in GSE167363.

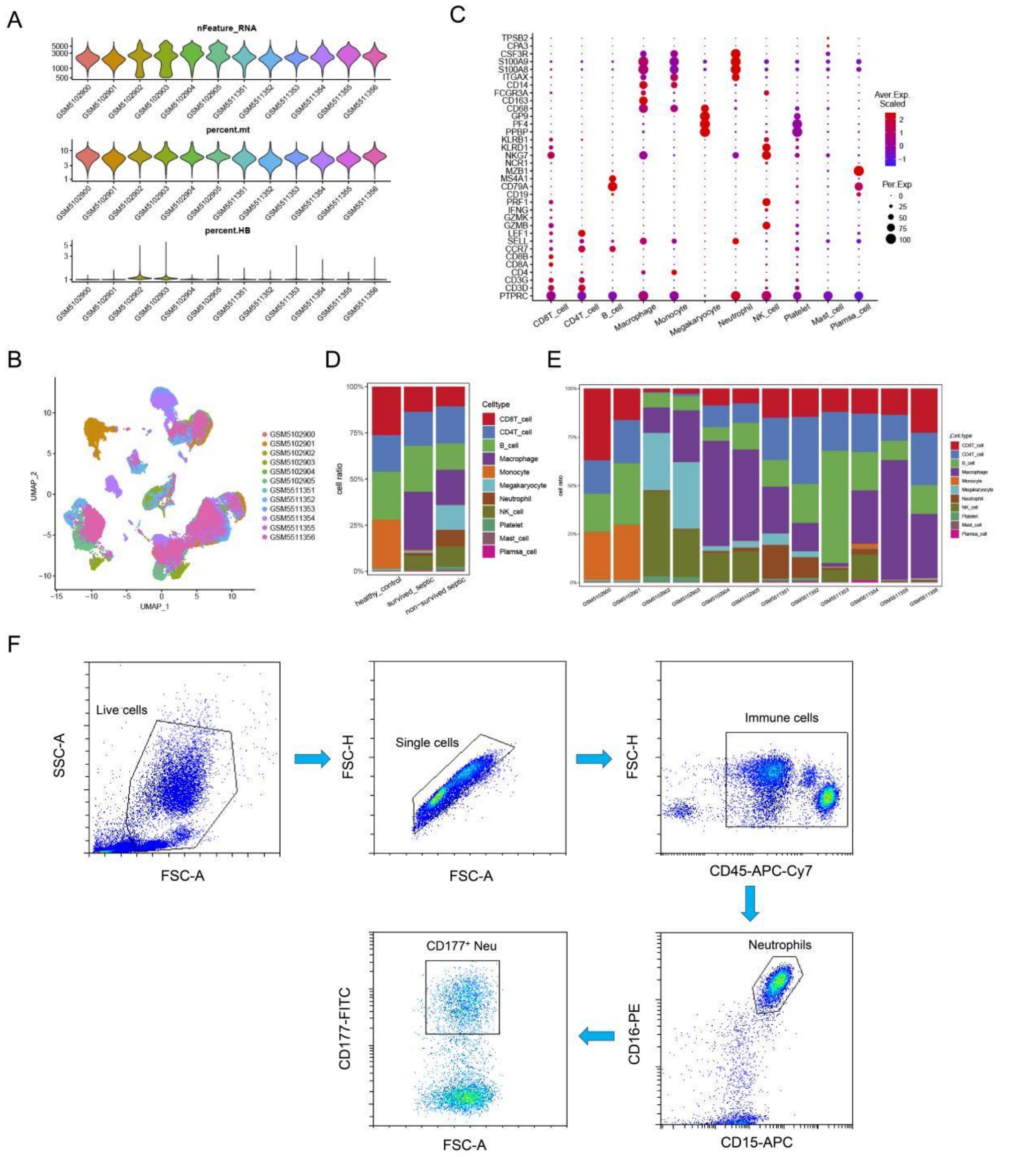

Supplementary Figure 4. Quality control, cell annotation, and flow cytometry gating strategy for single-cell and neutrophil analyses.

(A) Single-cell RNA-seq quality-control metrics, including RNA feature counts, mitochondrial gene percentage, and hemoglobin gene expression.

(B) UMAP visualization showing the distribution and mixing of cells from different patient samples after data integration.

(C) Bubble plot showing marker-gene expression patterns used for immune-cell annotation.

(D) Stacked bar plot showing immune-cell composition across healthy controls, surviving septic patients, and non-surviving septic patients.

(E) Individual-sample-level immune-cell composition across all analyzed participants.

(F) Flow cytometry gating strategy for neutrophil subset identification. $CD45^+$ leukocytes were first gated, followed by identification of $CD15^+CD16^+$ neutrophils and $CD177^+$ neutrophils based on the indicated surface markers.

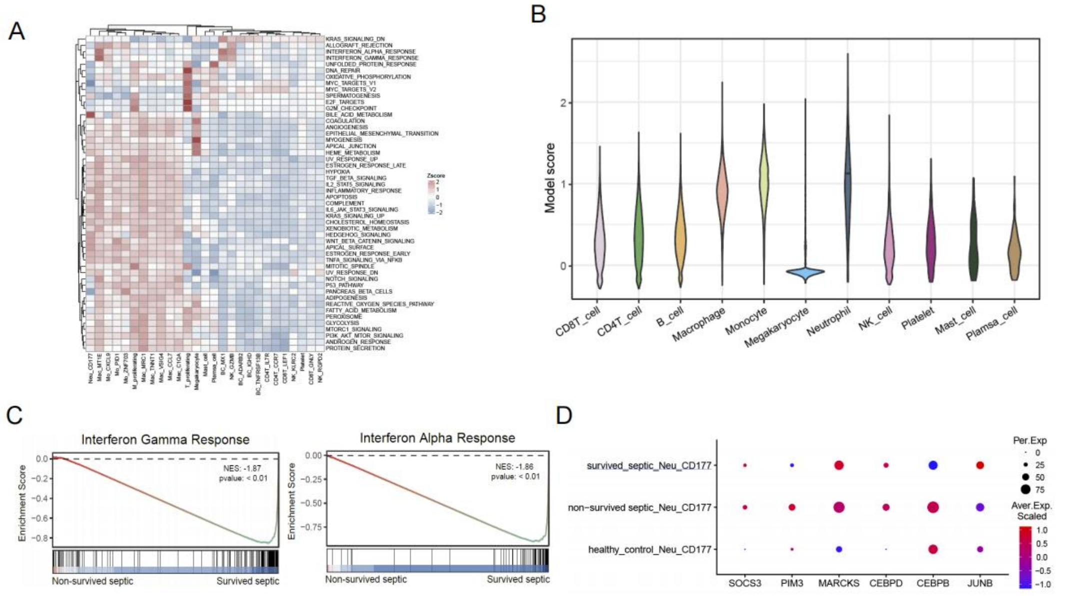

Supplementary Figure 5. Additional single-cell analyses of inflammatory pathway scores and Model-score gene expression in immune-cell populations.

(A) Hallmark pathway score analysis across immune-cell populations using AddModuleScore. The heatmap shows module scores for 50 Hallmark pathways across annotated immune-cell subsets, with red indicating higher scores and blue indicating lower scores.

(B) Distribution of the single-cell Model score across major immune-cell types. Violin plots show higher Model scores in neutrophils compared with other immune-cell populations.

(C) GSEA analysis of interferon-response pathways in Neu_CD177^{+} neutrophils. Running enrichment plots show lower enrichment of IFNγ and IFNα response pathways in neutrophils from non-surviving septic patients compared with surviving septic patients (IFNγ: NES = -1.87; IFNα: NES = -1.86; $p < 0.01$).

(D) Expression patterns of the six Model-score genes in Neu_CD177^{+} neutrophils across patient groups. Dot size indicates the percentage of expressing cells, and color indicates scaled expression level.

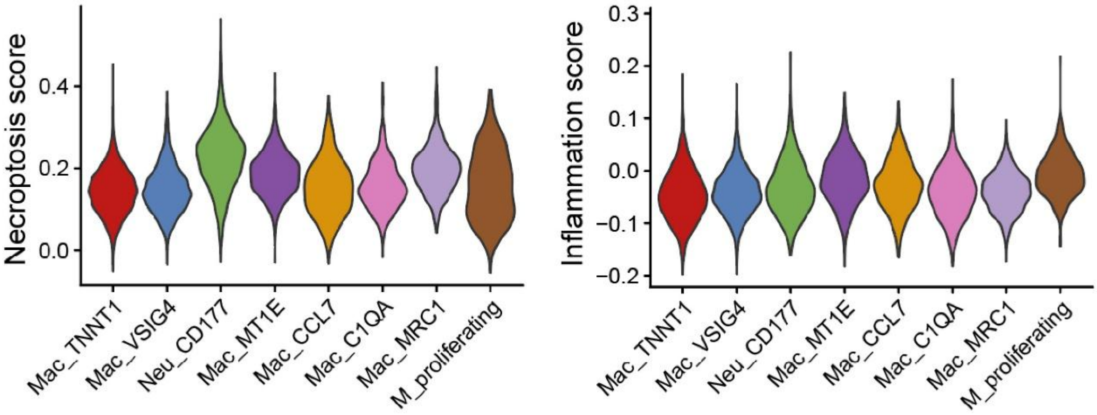

Supplementary Figure 6. Comparison of necroptosis-related gene-set scores and inflammatory-response scores across myeloid-cell subsets.

Distribution of necroptosis-related gene-set scores and inflammatory-response scores across myeloid-cell subsets. Necroptosis-related gene-set scores were calculated using the Necroptosis-1 gene set described in Supplementary Table 2. Inflammatory-response scores were calculated using the MSigDB HALLMARK_INFLAMMATORY_RESPONSE gene set as a reference inflammatory gene set.
